# Chemical Nature and Reaction Mechanisms of the Molybdenum Cofactor of Xanthine Oxidoreductase

**DOI:** 10.2174/1381612811319140010

**Published:** 2013-04

**Authors:** Ken Okamoto, Teruo Kusano, Takeshi Nishino

**Affiliations:** 1 Department of Biochemistry and Molecular Biology, Nippon Medical School, 1-1-5 Sendagi, Bunkyou-ku, Tokyo 113-8602; 2 Department of Applied Biological Chemistry, Graduate School of Agricultural and Life Sciences, University of Tokyo, 1-1-1 Yayoi, Bunkyo-Ku, Tokyo, 113-8657, Japan

**Keywords:** Xanthine oxidase, xanthine dehydrogenase, complex flavoprotein, molybdenum cofactor, uric acid, nitic oxide.

## Abstract

Xanthine oxidoreductase (XOR), a complex flavoprotein, catalyzes the metabolic reactions leading from hypoxanthine to xanthine and from xanthine to urate, and both reactions take place at the molybdenum cofactor. The enzyme is a target of drugs for therapy of gout or hyperuricemia. We review the chemical nature and reaction mechanisms of the molybdenum cofactor of XOR, focusing on molybdenum-dependent reactions of actual or potential medical importance, including nitric oxide (NO) synthesis. It is now generally accepted that XOR transfers the water-exchangeable -OH ligand of the molybdenum atom to the substrate. The hydroxyl group at OH-Mo(IV) can be replaced by urate, oxipurinol and FYX-051 derivatives and the structures of these complexes have been determined by x-ray crystallography under anaerobic conditions. Although formation of NO from nitrite or formation of xanthine from urate by XOR is chemically feasible, it is not yet clear whether these reactions have any physiological significance since the reactions are catalyzed at a slow rate even under anaerobic conditions.

## INTRODUCTION

Xanthine oxidoreductase (XOR) exists in a great variety of organisms from bacteria to higher plants and humans. The enzyme catalyzes the metabolic reactions leading from hypoxanthine to xanthine and from xanthine to urate, which are the last steps in the human purine excretion system. Thus, the enzyme is a target of drugs for therapy of gout or hyperuricemia [1]. Mechanistically, each enzyme reaction is an oxidative hydroxylation, and two electrons are passed from the substrate to the enzyme in each step [2]. Normally, NAD^+^ is the electron acceptor, forming NADH, but under certain conditions in mammals, conformational change of the enzyme protein is induced and the enzyme is transformed to xanthine oxidase (XO), of which the principal electron acceptor is molecular oxygen.

**Fig. (1):**
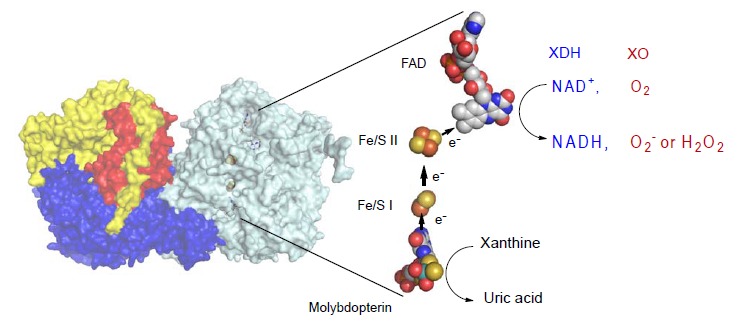
**Crystal Structure of Bovine XOR.** Left; Homodimer structure of bovine XOR. The N-terminal (in red), the C-terminal (in blue) and the intermediate (in yellow) domains contain the iron-sulfur centers, the molybdopterin and the FAD centers. Right: Cofactor arrangements of the enzyme. Figures were generated from PDB ID 1F4Q. Arrows show the directions of electron flow during catalysis. The reduced FAD reacts with either NAD^+^ or oxygen to produce NADH or hydrogen peroxide (H_2_O_2_) or superoxide (O_2_^-^). FADH_2_ reacts with O_2_ to produce H_2_O_2_ , while FADH produces O_2_^-^ [61, 63]. (The color version of the figure is available in the electronic copy of the article).

Mammalian XOR exists as a homodimer of 150 kDa subunits [3]. Each of the subunits is composed of three domains, as shown in (Fig. **1**). The largest domain contains the molybdenum center (molybdenum cofactor; molybdopterin), the intermediate contains flavin adenine dinucleotide (FAD) cofactor and the smallest contains the two iron sulfur centers ([2Fe-2S] type). The redox reaction centers are almost linearly positioned in the order of molybdopterin, two [2Fe-2S] type iron sulfur centers and FAD. The iron sulfur centers are called Fe/S I and Fe/S II, based on their redox potentials and EPR signals [4-9]; Fe/S II has the higher redox potential. Electrons that are passed to the molybdenum during the hydroxylation reaction are transferred to FAD via the iron sulfur centers. Finally, NAD^+^ or oxygen molecule, which is the final electron acceptor, is reduced.

 This enzyme is one of the best-studied complex flavoproteins and various reviews are available [10-17]. Here, we review the chemical nature and reaction mechanisms of the molybdenum cofactor of XOR, focusing on molybdenum-dependent reactions of actual or potential medical importance, including nitric oxide (NO) synthesis.

## I. PURINE HYDROXYLATION AS A MOLYBDENUM-DEPENDENT DEHYDROXYLATION REACTION

The hydroxylation reaction of purine occurs at the molybdenum cofactor [18]. In this cofactor, molybdenum binds with the pterin ring through two sulfur atoms. A further sulfur atom and two oxygen atoms are coordinated to the molybdenum, and are exposed to solvent [3, 19]. One of the oxygen atoms is derived from a water molecule, and is subsequently incorporated into the substrate [20, 21]. The general equation of the dehydrogenase reaction can be written as follows:

RH + OH^-^ = ROH + H^+^ + 2e^-^

where R may represent various nitrogen-containing cyclic molecules, such as hypoxanthine and xanthine, which are the physiological substrates of the enzyme, as well as aldehydes. As a result of this reaction, the enzyme receives H^+^ + 2e^-^ from the substrate (that is to say, the enzyme is reduced), and this is called the reductive half-reaction. On the other hand, the process of transfer of an electron from the enzyme to NAD^+^ or O_2_ is called the oxidative half-reaction. The next two equations show the scenario in which oxygen (O_2_) serves as a substrate in the xanthine oxidation reaction.

Eox + Xanthine → Ered + urate 

Ered + O_2_ → Eox + H_2_O_2_ (+ O_2_^-^)

This is different from the so-called oxygenase reaction [22] discovered by Hayaishi *et al*., but is a classical example of dehydrogenation. That is, as mentioned above, an electron is removed from the substrate and an oxygen atom originating from a water molecule (OH^-^) is taken into the substrate [20, 21]. On the other hand, in an oxygenase reaction, the substrate receives an electron from a donor such as NADPH and an oxygen atom derived from molecular oxygen is transferred to the substrate.

 Bray *et al*. first showed that the hydroxylation reaction was dependent on molybdenum by ESR observation of the Mo signal after trapping the reaction intermediate by means of a rapid freezing method [23]. Firstly, two electrons are transferred from the substrate to Mo, reducing Mo(VI) to Mo(IV); Mo(V) is observed only transiently. The actual involvement of Mo(V) formation has been confirmed only quite recently. It is considered that H^+^+ 2e^-^ derived from the substrate, in the form of hydride (H^-^), i.e. a hydrogen atom with two electrons, is transferred to a sulfur ligand of the molybdenum atom (Mo(VI)=S → Mo(IV)-SH) [17, 18, 21]. The Mo(V) species is not formed by one-electron transfer from the substrate, but rather is formed during the process of electron transfer from Mo to the iron sulfur center. This will be further discussed in part IV.

## II. METABOLIC HYDROXYLATION OF HYPOXANTHINE TAKES PLACE AT THE 2-POSITION INITIALLY, THEN AT THE 8-POSITION

**Fig. (2):**
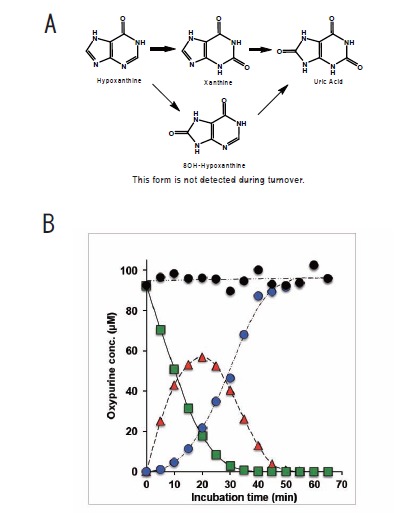
**Hydroxylation Reaction of Purines. A;** Hydroxylation order in purine catabolism. Accumulation of xanthine was observed, indicating that the reaction does not occur sequentially. The absence of formation of 6, 8-dihydroxypurine suggests that hydroxylation at the 2-position is important for hydroxylation at the 8-position. **B;** Concentrations of hypoxanthine, xanthine and urate were measured with HPLC during turnover from hypoxanthine to urate catalyzed by bovine milk xanthine oxidase. (T. Kusano, unpublished results).

In the purine catabolic pathway, hydroxylation of hypoxanthine (6-hydroxypurine) initially takes place at the 2-position, yielding xanthine (2,6-dihydroxypurine). The next hydroxylation occurs at the 8-position, affording urate (2,6,8-trihydroxypurine) (Fig. **2A**). This sequence was predicted from spectral observation [24, 25], and it was confirmed by means of time-resolved spectroscopy that when purified XOR and hypoxanthine were mixed under aerobic conditions, hydroxylation proceeded in the order 2-position → 8-position [26]. (Fig. **2B**) shows the accumulation of xanthine directly observed after separation on a HPLC column. During the reaction, xanthine accumulates and is then transformed into urate. This fact suggests that the hydroxylation at the 2-position influences the subsequent hydroxylation at the 8-position of 6-hydroxypurine, and this will be further considered during the discussion of the substrate activation mechanism and xanthine binding mode in part IV. It is important to note that when XOR activity is measured in terms of UV absorbance changes, xanthine, not hypoxanthine, is generally used as a substrate; since urate production does not occur in a single step from hypoxanthine, it is impossible to precisely measure the initial velocity. On the other hand, 6-amino-8-hydroxypurine is detected in urine of patients with adenine phosphoribosyltransferase (APRT) deficiency; this finding indicates that hydroxylation of adenine occurs at the 8-position prior to the 2-position, though the reaction is extremely slow [27].

## III. ELECTRONS ARE TRANSFERRED QUICKLY TO ENZYME COFACTORS. RATE-LIMITING STEP IN THE STEADY STATE IS THE DISSOCIATION OF URATE

　Electrons transferred to molybdenum are quickly distributed to other reaction centers [28]. Before the crystal structure was elucidated, the electron transfer pathway was unclear, but stopped-flow and steady-state kinetic studies have shown that electron transfer in the molecule is very rapid, and in the steady state the overall reaction with O_2_ or NAD^+^ can be considered as two half reactions, as discussed in section II.

Fig. (**3**) shows the reaction rate at each step of the xanthine-O_2_ reaction observed by using stopped-flow methods at low temperature. The slowest step, i.e. the rate-determining step, is the dissociation of urate [29]. This is very important when discussing the reaction mechanism in relation to the protein structure. The electron transfer pathway within the enzyme molecule is Mo →Fe/S(I) →Fe/S(II) →FAD, and pulse radiolysis analysis confirmed that the overall process is very rapid [30, 31]. The stopped-flow method takes milliseconds to mix the enzyme and substrate, so it is impossible to observe the electron transfer phase, but in pulse radiolysis, electrons are directly introduced and reactions can be measured on a timescale of microseconds. Electrons transferred to FAD are finally passed to NAD^+^ or oxygen (O_2_), yielding NADH, hydrogen peroxide or superoxide. 

## IV. PURINE BINDING MODE AND REACTION MECHANISM

Although various models of the hydroxylation mechanism of XOR have been proposed in recent years based on ESR studies and model complexes, X-ray crystallography and analysis of point-mutated enzymes have allowed a definitive conclusion to be reached regarding the hydroxylation mechanism of purine bases. According to the X-ray structure of salicylic acid (a weak inhibitor that competes with xanthine) complex with XOR, the benzene ring of salicylic acid is bound between two phenylalanines (Phe914 and Phe109 in the bovine sequence), which are conserved in all organisms so far examined [2, 32]. It is presumed that the purine ring of substrates (xanthine and hypoxanthine) is similarly fixed between these two phenylalanines [3].

**Fig. (4):**
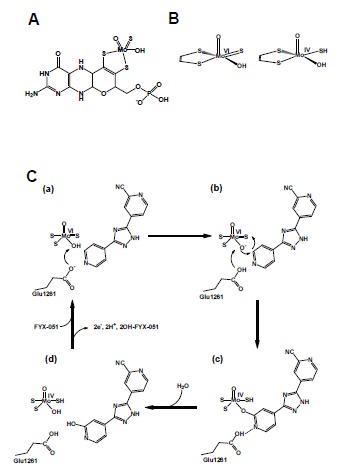
**Structure of Mammalian Molybdopterin and Hydroxylation Mechanism of Artificial Slow Substrate FYX-051. A;** Chemical structure of mammalian XOR molybdopterin. **B;** Geometry of molybdenum-coodinated atoms in the oxidized state (*left*) and reduced state (*right*). **C;** Glu1261 works as a base, abstracting the proton from Mo-OH (**a**). The generated Mo-O- nucleophilically attacks the carbon center to be hydroxylated, with concomitant hydride transfer (**b**). The protonated Glu1261 forms a hydrogen bond to the N1-nitrogen of the substrate, and this facilitates the nucleophilic attack (**b**). The reduced Mo(IV) coordinated to the product via the newly introduced hydroxyl group (**c**). The intermediate breaks down by hydroxide displacement of the product (**d**).

 The structure of the molybdenum center is illustrated in (Fig. **4A**). It is now generally accepted that XOR transfers the water-exchangeable -OH and not the Mo=O ligand of molybdenum to the substrate [33, 34]. The free electron pairs of oxygen then attack the electrophilic carbon to yield the hydroxylated product. Its equatorial location appropriately positions the Mo=S ligand to accept the hydride ion from the reactive carbon atom, affording Mo-SH and a reduced molybdenum center (Fig. **4B**). 

The geometry of the molybdenum-coordinated atoms has been elucidated through X-ray crystal structure analysis of the complex with FYX-051, which is an inhibitor introduced by Fuji Yakuhin Co. A sulfur atom and water-derived oxygen atom were found to be coordinated to the equatorial plane of the molybdenum atom (Fig. **4C**), and this geometry is consistent with magnetic circular dichroism findings [35], X-ray absorption spectroscopy [36] and analysis of model compounds [37]. The inhibitor is a slowly reacting substrate and forms a quite stable reaction intermediate with the molybdenum center. The X-ray crystal structure revealed the presence of a Mo-O-C bond [19]. The role of two glutamate residues in the vicinity of molybdenum was indicated by an analysis of mutated enzymes [38, 39]. Protonated Glu1261 forms a hydrogen bond with substrate nitrogen, facilitating nucleophilic attack on adjacent carbon by the basic oxygen atom (Mo-O^-^) [19, 38]. This concept is also consistent with the role of the side chain of a glutamic acid residue near the Mo-OH group in reaction of aldehyde oxidoreductase (ALO) from the sulfate-reducing anaerobic bacterium *Desulfovibrio gigas *[40]. When that residue was altered by mutagenesis to alanine, ALO was completely inactivated.

**Fig. (5):**
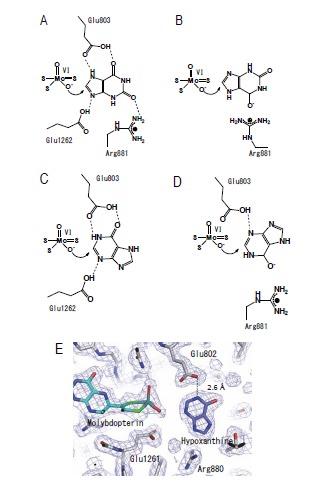
**Two Hydroxylation Models of Xanthine Hydroxylation. A;** Proposed model of xanthine binding mode based on the analysis of mutant enzymes, as well as the urate binding mode. The hydrogen bonds of the three amino acids promote nucleophilic reaction at C8 (based on 38, 45). **B;** Activation of substrate xanthine by Arg881 via accumulation of negative charge at the 6-position oxygen (based on 39). **C;** Proposed hypoxanthine binding mode based on the analysis of mutant enzymes (based on 38) and binding mode of hypoxanthine to the desulfo-form in the crystal (Fig. **5E**). **D;** Activation of substrate hypoxanthine owing to accumulation of negative charge at the 2-position oxygen. The crystal structure of a different binding mode from C was also reported (based on 42). **E;** Crystal structure of hypoxanthine bound bovine desulfo-XOR, which lacks an essential sulfur atom at the active site, at 2.0 Å resolution (unpublished data). The 2Fo-Fc electron density map was contoured at 1.3 σ. A hydrogen bond is shown as a broken line.

**Fig. (6):**
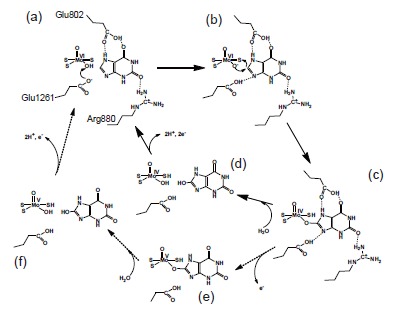
**Proposed Hydrogen-Bonding Arrangement of the Xanthine-bound Complex with Molybdopterin, and Mechanism of the Xanthine Hydroxylation Based on this Binding Mode.** Glu1261 abstracts the proton from Mo-OH (**a**). The -O^-^ thus generated is then involved in electrophilic attack on the C8 carbon of xanthine with hydride transfer to the =S of the molybdopterin (**b**), resulting in a covalent linkage between the molybdenum ion and the C8 carbon atom via the bridging oxygen atom (**c**). The protonated Glu1261 and glutamate Glu802, which is also supposed to be protonated under physiological conditions, form hydrogen bonds to the substrate, stabilizing this state. Arg880, too, is involved in the hydroxylation by forming a hydrogen bond with the O2 atom of the xanthine molecule. The intermediate decomposes via the replacement of the bridge oxygen with a water molecule (**d** or **e~ f**).

**Fig. (7):**
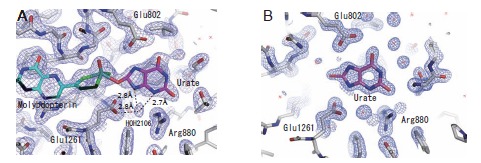
**Binding Modes of Urate with the Demolybdo Form of the Enzyme. A;** The structure of the complex of urate bound to the reduced bovine XOR under anaerobic conditions was also determined. The 2F_o_-F_c_ electron density map contoured at 1.0 σ. **B;** As urate dissociates from the holoenzyme without forming a stable binding mode, the X-ray crystal structure of the urate-bound form of rat XOR D428A mutant enzyme without the molybdenum cofactor (demolybdo enzyme) was determined. The 2F_o_-F_c_ electron density map was contoured at 1.5 σ. Figures were generated from PDB ID 3AMZ and 3AN1, respectively (45).

Regarding the role of charged residues at the active center, Yamaguchi *et al*. found that the enzyme activity was decreased significantly upon mutation of Glu803 and Arg881 (Glu802 and Arg880, respectively, in the bovine enzyme). Proposed binding modes of substrate hypoxanthine and xanthine (Fig. **5A** and **C**) suggested that the nucleophilic reaction is activated or facilitated through hydrogen bonds formed between the substrate and these amino acid residues [38]. The hydroxylation mechanism based on this model is summarized in (Fig. **6**). These binding modes are consistent with the metabolic sequence, i.e., hydroxylation at the 2-position of hypoxanthine precedes that at the 8-position. That is, the interaction of the 2-position keto group (C=O) and Arg881 is important for efficient hydroxylation at the 8-position. This is consistent with the report that mutation of the corresponding arginine to glutamate in *A. nidulans* XOR resulted in loss of xanthine hydroxylation activity, though activity towards hypoxanthine still remained [41]. Although another binding mode and activation mechanism have been proposed (Fig. **5B** and **D**) [39, 42], a QM/MM study supported the model shown in (Fig. **4A**) [43, 44], and an X-ray crystallographic study of the urate-bound reduced enzyme showed a similar binding mode to that in (Fig. **4A**) (Fig. **7**) [45]. We also determined the crystal structure of hypoxanthine bound form to the desulfo-XDH (5E), consistent with the mechanism proposed (38). Although Glu802 was proposed to promote tautomerization of xanthine in the alternative binding mode, it should be noted that the electron density of a water molecule was clearly observed. This water molecule, located at 3N and 9N of xanthine (Fig. **7A**, HOH2106), may serve to assist release of the urate product [2, 43]. 

## V. COVALENT LINKAGES BETWEEN REDUCED MOLYBDENUM AND INHIBITORS

**Fig. (8):**
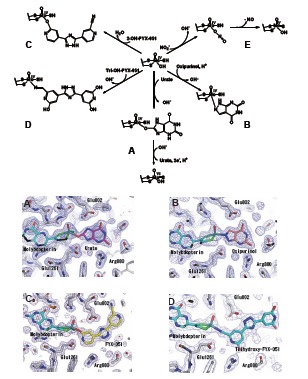
**Summary of the Chemical Structures of Ligands Covalently Bound to Reduced Molybdenum in the Active Site of XOR. A;** urate, **B:** oxipurinol, **C:** FYX-051, **D:** trihydroxy-FYX-051. **E;** proposed mechanism of NO formation by XO based on reference [57]. Related crystal structures are shown below. Figures were generated from PDB ID 3AMZ, 3BDJ, 1V97 and 3AM9, respectively (45, 51, 19, 52).

Inhibitors of XOR are used as antigout drugs. Some compounds inhibit the enzyme by forming a stable reaction intermediate or analog of this intermediate with reduced molybdenum. Allopurinol (4-OH-pyrazolo-pyrimidine), which has been used as an anti-gout drug for over 40 years, is an isomer of hypoxanthine in which the 8-position carbon atom is replaced with the adjacent 7-position nitrogen atom. It was initially thought to work as a simple competitive inhibitor that binds to the molybdenum center competitively with respect to xanthine [46]. However, subsequently it was found that the inhibitory mechanism of allopurinol is more complicated; the inhibition progresses time-dependently and finally a tightly bound complex of the reduced enzyme is formed, *i.e.* allopurinol itself is a substrate of XO and is transformed to oxipurinol (alloxanthine; 4,6-dihydroxypyrazolopyrimidine), which forms a covalent bond with Mo(IV) (Fig. **8B**) [47-49]. In the crystal structure, the exchangeable oxygen of Mo(IV) is replaced by the nitrogen atom at the 2-position (corresponding to the 8-position of the purine base) of oxipurinol [50, 51]. The formation of this oxipurinol-molybdenum complex is the reason for the potent inhibitory effect of allopurinol, though the complex dissociates upon reoxidation of Mo(IV) in air (t_1/2_=300 min at 25˚C) [49].

FYX-051 is an artificial substrate of XOR [11]. FYX-051 transfers two electrons to the enzyme, forming a stable complex with Mo(IV) (Fig. **8C**). In the crystal structure, it forms covalent bonds with molybdenum through oxygen atoms derived from the substrate. In addition, various interactions, including hydrogen bonding with surrounding amino acids, hydrophobic interaction and π-π stacking interaction, have been observed. Hydroxy-FYX-051-Mo(IV) is subject to natural re-oxidation of Mo(IV) in air, but its half-life at 25°C is approximately 20 hours. Determination of the crystal structure of the actual reaction intermediate, i.e., the XOR and FYX-051 complex, provided important insight into the hydroxylation mechanism (Fig. **4C**). An identical structure was also formed when dithionite-reduced Mo(IV) was mixed with hydroxy-FYX-051, resembling the urate-bound form described above. When the FYX-051-XOR complex was incubated for a longer period (5 days) under anaerobic conditions, a significant increase in its absorbance near 600 nm was observed and it was assumed that this was due to formation of trihydroxy-FYX-051 and XOR complex [52]. This complex was observed by means of X-ray crystal structure analysis. It was found that trihydroxy-FYX-051 forms a covalent bond with molybdenum through a nitrile group (Fig. **8D**) and they are linked through a different bond from that in the reaction intermediate formed in the initial stage. In the structure, the exchangeable hydroxyl group of Mo(IV) is replaced by the N atom of the CN group of the inhibitor [52].

## VI. NO FORMATION FROM NITRITE AT THE MOLYBDENUM SITE

Recently, several groups have reported that XOR, including mammalian enzymes, can reduce nitrite to yield nitric oxide (NO) [53-57], which is a physical vasodilator. XO reduces nitrite at molybdenum and produces NO under hypoxic conditions with NADH as an electron donor (Fig. **8E**), and the XOR-produced NO may play a protective role in myocardial ischemia [58, 59].****It has been argued that this NO production occurs *in vivo* as part of a defense or regulatory response under some pathogenic conditions, such as tissue ischemia [60]. However, it should be noted that the reported NO production by XO is very low even under anaerobic condition: *Kcat* is 0.17 s^-1^ and *Km* for NADH is ~ 0.9 mM at 37 ˚C [56]. These values correspond to only about ~1% of urate formation activity from xanthine at lower temperature (~15-20 s^-1^at 25˚C) [61, 62], likely because the arrangement of the cofactors is not thermodynamically favorable (NADH reacts with higher potential at FAD, while the NO formation site is at lower potential molybdenum couples). Somewhat higher values were reported with xanthine or aldehyde as another substrate [56]: *Kcat* is 0.68 s^-1^ and *Km* 1.46 µM for xanthine substrate, while *Kcat* is 0.34 s^-1^ and *Km* 35 µM for 2,3-dihydroxybenzaldehyde. Maia *et al*. reported similar values for aldehyde substrates [57]. It is not surprising from a chemical point of view that the water-exchangeable hydroxyl group at OH-Mo(IV) can be replaced by NO_2_ to produce NO, since various compounds, such as urate (**8A**), oxipurinol (**8B**) and FYX-051 derivatives (**8C**,** 8D**), can behave similarly, as mentioned above. It is also true that XOR produces xanthine from urate under certain conditions (e.g, strictly anaerobic conditions), albeit with very low activity. It is possible that urate could be a competitor of NO_2_, since urate concentration in tissue is relatively high (estimated to be ~ 50 µM in moderate hyperuricemia). Although formation of NO from nitrite or formation xanthine from urate by XOR is chemically feasible, it is not yet clear whether these reactions have any physiological significance.

## Figures and Tables

**Fig. (3):**
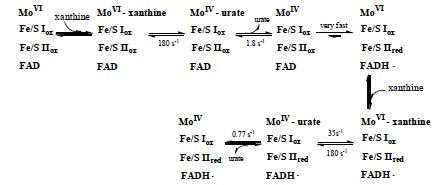
**Schematic Diagram of the Reaction of Molybdenum during Xanthine Hydroxylation.** The results of fast reaction between xanthine and chicken xanthine dehydrogenase followed by means of a stopped-flow method at 4°C. Dissociation of urate (1.8/sec) is the slowest step, and is rate-limiting for the overall process. This figure is drawn based on reference [29].
